# Clinical Experience of High Frequency and Low Frequency TENS in Treatment of Diabetic Neuropathic Pain in Russia

**DOI:** 10.3390/healthcare10020250

**Published:** 2022-01-28

**Authors:** Mustafa Al-Zamil, Inessa A. Minenko, Natalia G. Kulikova, Michael Alade, Marina M. Petrova, Elena A. Pronina, Irina V. Romanova, Ekaterina A. Narodova, Regina F. Nasyrova, Natalia A. Shnayder

**Affiliations:** 1 Department of Physiotherapy, Faculty of Continuing Medical Education, Peoples’ Friendship University of Russia, 117198 Moscow, Russia; www.kulikova@rambler.ru; 2 Department of Sports Medicine and Medical Rehabilitation, Sechenov First Moscow State Medical University, 119435 Moscow, Russia; kuz-inna@mail.ru; 3 Well Street Surgery, London E9 7TA, UK; m.alade@nhs.net; 4 Shared Core Facilities “Molecular and Cell Technologies”, V. F. Voino-Yasenetsky Krasnoyarsk State Medical University, 660022 Krasnoyarsk, Russia; stk99@yandex.ru (M.M.P.); diinny@mail.ru (E.A.P.); irinavr2018@gmail.com (I.V.R.); katya_n2001@mail.ru (E.A.N.); 5 Institute of Personalized Psychiatry and Neurology, V.M. Bekhterev National Medical Research Centre for Psychiatry and Neurology, 192019 Saint Petersburg, Russia; nreginaf77@gmail.com; 6 International Centre for Education and Research in Neuropsychiatry, Samara State Medical University, 443099 Samara, Russia

**Keywords:** neuropathic pain, transcutaneous electrical nerve stimulation, TENS, high frequency, low frequency, VAS, distal polyneuropathy, MPQ, Pain Drawing

## Abstract

Background: Transcutaneous electrical nerve stimulation (TENS) is presently one of the main methods of treatment for neuropathic pain in type II diabetes mellitus. The discussion about which TENS frequency is more effective in the treatment of neuropathic pain has been ongoing for many years. Despite this, the response of different aspects of neuropathic pain to various TENS modalities has not been sufficiently studied. Aim: To analyze changes in characteristics of neuropathic pain depending on the frequency of TENS. Materials and methods: Seventy-five Russian diabetic patients with painful distal axonal neuropathy were enrolled in the study. Patients were assigned to three groups: in the HF TENS group, 25 patients received standard drug therapy (Alpha-lipoic acid, Pentoxifylline, Vitamin B12, Gabapentin) + high-frequency TENS (HF); in the LF TENS group, 25 patients received standard drug therapy (Alpha-lipoic acid, Pentoxifylline, Vitamin B12, Gabapentin) + low-frequency TENS (LF); in the control group, 25 patients underwent just standard drug therapy (Alpha-lipoic acid, Pentoxifylline, Vitamin B12, Gabapentin). Pain intensity was calculated before and after treatment with visual analogue scale (VAS), McGill pain questionnaire (MPQ), Douleur Neuropathique 4 Questions (DN4) and Pain Drawing. Results: TENS increased the therapeutic effect of standard drug therapy, in the treatment of neuropathic pain, by 65.9% and prolonged its efficacy by 31% for up to 6 months after treatment. HF TENS had a more pronounced analgesic effect than LF TENS based on VAS (34.7%), sensory (57.6%) MPQ dimensions and DN4 (21%). Affective MPQ dimension with the use of LF TENS was lower than HF TENS by 34.7% immediately after treatment, by 47.3% after 2 months and by 34.8% after 6 months of the follow-up period. Conclusion: There are significant differences between HF and LF TENS based on pain assessment using various pain scales. This reflects the distinctive effects of different TENS modalities on different aspects of neuropathic pain.

## 1. Introduction

The Transcutaneous Electrical Nerve Stimulation (TENS) of peripheral nerves for treatment of diabetic distal polyneuropathy in Russia was used for the first time in 2015 [1]. By 2015, TENS efficacy in the treatment of peripheral nervous system pathology was considered proven, based on several mechanisms of action:

- Regional improvement of blood circulation in nerve and surrounding tissue [2,3];
- Increased axonal transport, contributing to the accumulation of growth factors in peripheral nerves [4,5,6];
- Creation of anastomoses between intact and damaged peripheral nerve fibers [7];
- Reinnervation of muscle fibers [4];
- Regeneration of peripheral nerve fibers [6,8,9];
- Inhibition of pro-inflammatory cytokines [2].

Previous studies demonstrated that direct TENS enhances the efficacy of standard drug therapy in the treatment of diabetic distal neuropathy by reducing negative sensory dysfunction by 1.5 times, positive sensory dysfunction by 2.1 times and neuropathic pain in 40% [1,10,11].

We additionally found evidence that lower limb distal neuropathy symptom resolution depends on the modality of applied electrical impulses. Positive sensory symptoms respond 23.9% better to high-frequency TENS (HF) compared to low-frequency TENS (LF), while the difference of negative sensory symptom decrease between LF TENS and HF TENS was 37.9%. However, existing clinical and experimental research dedicated to the study of direct TENS has not fully investigated the changes in characteristics of neuropathic pain depending on TENS frequency and amplitude [7,12].

It is important to note that previous studies attempted to carry out a comparative analysis between indirect HF TENS (frequency from 100 to 200 Hz) and LF TENS (1–3 Hz) [7,13]. However, these studies lacked uniform criteria, used multiple TENS techniques, different generated currents and enrolled patients with neuropathic pain of various etiology [7]. In addition, pain intensity assessments were carried out using a single scale, which was the visual analogue scale (VAS) in the majority of cases.

The aim of this study was the analysis of changes in characteristics of neuropathic pain depending on the TENS frequency.

## 2. Materials and Methods

### 2.1. Participants

We enrolled 75 (female 38, male 37) adult Russian patients with type II diabetes mellitus complicated by lower limb painful distal axonal neuropathy.

**Figure 1:**
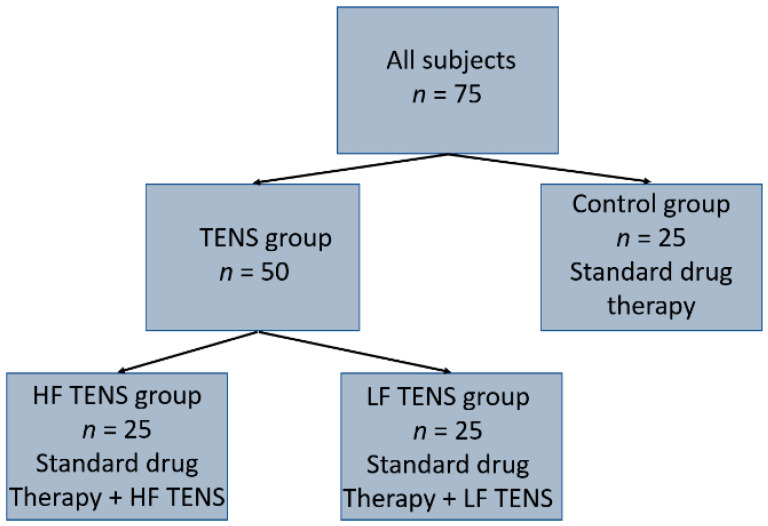
Distribution of patients into groups.

The control group (*n* = 25; female 13, male 12) received only standard drug therapy. In addition to standard drug therapy, the TENS group (*n* = 50; female 25, male 25) was managed with TENS. Depending on the form of TENS, patients were divided into two groups: the HF TENS group (*n* = 25, female 12, male 13) underwent a course of HF TENS, and the LF TENS group (*n* = 25, female 13, male 12) completed a course of LF TENS (Figure 1).

The mean age of the studied patients was 39 ± 2 years old. The control group included patients aged from 25 to 54 years old, mean age 39.5 ± 2 years old. HF TENS group included patients aged from 24 to 53 years old, mean age 39 ± 2 years old. LF TENS group included patients from 25 to 54 years old, mean age 39.8 ± 2 years old. From these data, it can be noted that there is no significant difference between mean age values in the studied groups (*p* > 0.05).

The duration of type II diabetes mellitus was 9.3 ± 0.46 years in the control group, 9.8 ± 0.36 years in the HF TENS group and 9.6 ± 0.36 years in the LF TENS group. The mean serum level of glycated hemoglobin (HbA1c) was 6.7 ± 0.12% in the control group, 6.8 ± 0.13% in the HF TENS group and 6.7 ± 0.12% in the LF TENS group.

Inclusion criteria in the TENS and control groups:

- Age—from 21 to 60 years old, including first period middle-aged (males 22–35 years, females 21–35 years) and second period middle-aged (males 36–60 years, females 36–55 years);
- Medically stable type 2 diabetes mellitus (the diagnosis was made by an endocrinologist or internist);
- Serum level HbA1c in two separate tests is lower than 7.0%;
- Distal axonal neuropathy of lower extremity vitrificated by electromyography (EMG);
- History of neuropathic pain for over 6 months;
- Neuropathic pain intensity is 5 scores or more on the visual analogue scale (VAS) and 4 scores or more on the Douleur Neuropathique 4 Questions (DN4).

Exclusion criteria in the main and control groups:

- Epilepsy and uncontrolled seizure disorder;
- Severely cognitive disorders;
- Mental illness;
- Hereditary polyneuropathy;
- History of peroneal nerve injury in the fibular tunnel or tibial nerve in the tarsal tunnel;
- History of stroke, spinal cord injury, traumatic brain injury, multiple sclerosis;
- Edema of lower extremity;
- History of cardiac arrhythmias or hemodynamic instability;
- Cardiac pacemaker or other implanted electronic system;
- Botulinum toxin injections to any lower extremity muscle in the last 3 months;
- Evidence of deep venous thrombosis or other forms of venous thromboembolism;
- Rheumatoid arthritis, gout, psoriasis and arthrosis of the joints of the lower extremities;
- Vascular atherosclerosis of the lower extremities.

Voluntary Informed Consent in Research and Clinical Care was signed by all patients. After an explanation of the medical condition, the purpose and benefits of the test, procedure or treatment and description of the proposed test, procedure or treatment were analyzed, including possible complications or adverse events. The study protocol was approved by the local medical ethics committee (protocol № 34, 23.01.2019). All procedures adhered to the 1984 Declaration of Helsinki and its later amendments. All patients read the abovenamed article in full (including text, figures and Supplementary Material) and agree to its publication.

Participation is not rewarded in this study. The researchers were not rewarded for their work. The study was carried out as part of a scientific research program of the Department of Physiotherapy of Peoples’ Friendship University of Russia.

### 2.2. Characteristics of Standard Drug Therapy

All patients received identical drug therapy with Alpha-lipoic acid 600 mg/day for 2 months: Pentoxifylline 100 mg t.i.d. during 1 month per orally; Vitamin B12 1000 mcg in muscular s.i.d during 10 days. Neuropathic pain was treated with Gabapentin 300 mg t.i.d. for 2 months orally.

### 2.3. Characteristics of Motor and Sensory Disorders

Negative sensory symptoms of distal axonal neuropathy scores were based on the prevalence of symptoms on a 5-point scale. The mean scores in the control group were 2.7 ± 0.17 points, 2.8 ± 0.16 points in the HF TENS group and 2.8 ± 0.17 points in the LF TENS group, *p* > 0.05. Positive sensory symptoms of distal axonal neuropathy were determined by patients (using a 10-point scale). The mean scores in the control group were 6.8 ± 0.27 points, 6.8 ± 0.26 points in the HF TENS group and 6.9 ± 0 points in the LF TENS group, *p* > 0.05.

Foot dorsiflexion paresis was diagnosed in 4 cases in the control group (strength on a 5-point scale: 3.5, 4.0, 3.0, 3.5 points), 5 cases in HF TENS group (3.5, 3.0, 3.0, 4.0, 4.0 points) and 4 cases in LF TENS group (3.0, 3.0, 3.5, 4.0 points), *p* > 0.05. Paresis of foot plantar flexion was diagnosed in 1 case in control group (4.0 point), 1 case in HF TENS group (4.5 points) and in 2 cases in LF TENS group (4.0 point), *p* > 0.05.

### 2.4. Characteristics of Neuropathic Pain Assessment Tools

Visual analogue scale (VAS): Patients were asked to rate their pain on a scale of 0 to 10 points.

McGill pain questionnaire (MPQ): Pain characteristics were assessed using MPQs. Patients self-reported their experience of pain in the sensory, affective and evaluative questionnaire dimensions. The questionnaire contains 78 words serving as pain descriptors, grouped into 20 categories and further divided into 3 dimensions: sensory (1–14), affective (15−19) and evaluative (20). The evaluative scale consists of 5 words expressing a generalized subjective assessment, similar to the usual verbal scale. Each category contains descriptors arranged in ascending order of synonymous meaning. The rank value for each descriptor is based on its position in the word set. The sum of the rank values is the pain rating index (PRI).

Pain Drawing: Location and distribution of pain were assessed using the Pain Drawing Method. Patients indicated the location of their pain on human drawings. Pain projection area (PPA) was determined by using a special two-dimensional transparent mesh.

DN4: Neuropathic pain was differentiated from nociceptive and psychogenic pain by the DN4 questionnaire.

Patients were assessed before, on completion and after completion of treatment. The post-treatment follow-up period was carried out 2, 4 and 6 months after completing therapy with and without TENS. 

### 2.5. TENS Technique

TENS was applied with a CE0434 certified BTL-4000 smart/premium device (BTL Industries Ltd., Hertfordshire, UK). Registration number P3H 2020/12648 dated 24 November 2020. The device has been approved in Russia since 2010 by registration certificate number ΦC3 2010/06686 dated 29 April 2010.

Protocol of HF TENS: We used monophasic impulses with a frequency of 100 Hz and a duration of 100 μs. Impulse amplitude was adjusted individually, from 5 to 20 mA, to achieve painless sensory sensations. Peroneal and tibial nerves were stimulated for 5 min each. There was a total of 15 sessions carried out on alternate days [1,10,11].

**Figure 2:**
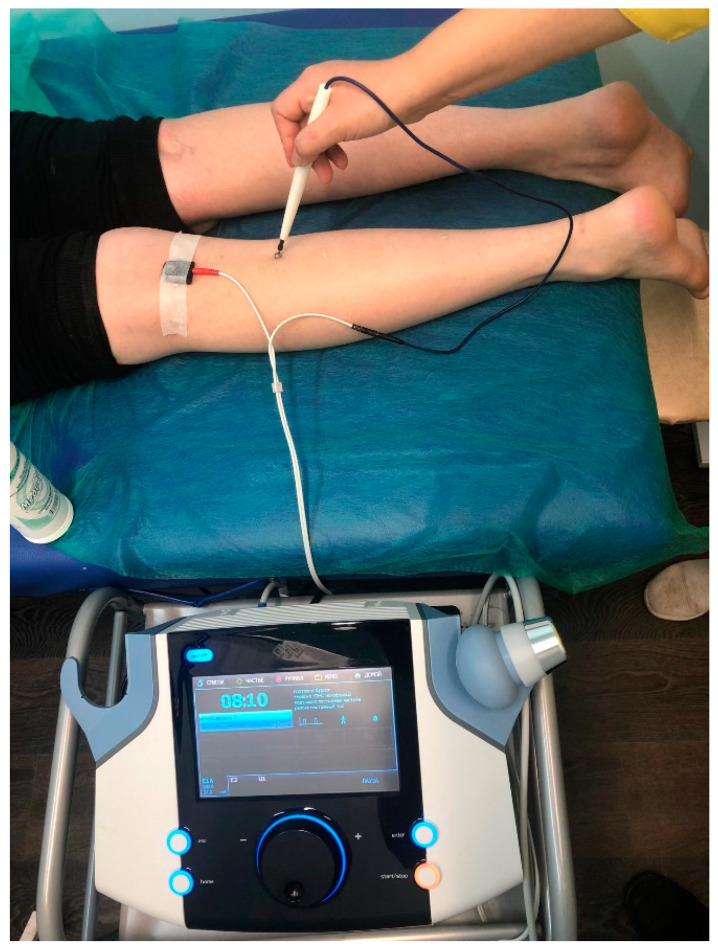
Stimulation of *n. tibialis* by LF TENS: cathode was fixed above the nerve in the popliteal fossa; pen-like anode was lable and moved from the proximal nerve to the distal direction.

Protocol of LF TENS: Monophasic impulses were used with a frequency of 1 Hz and duration of 200 μs. Impulse amplitude was individually adjusted from 5 to 40 mA until painless muscle contraction was achieved. Peroneal and tibial nerves were stimulated for 5 min each (Figure 2). There was a total of 15 sessions carried out on alternate days [1,10,11].

### 2.6. Statistical Analysis

Collected data were computed and analyzed using SPSS software for Windows, version 20 (IBM, Armonk, NY, USA). 

Descriptive statistics were used to describe the mean ± standard error of the mean (mean ± S.E.M.) of the participants’ characteristics. Normality was tested using Shapiro–Wilk normality test. Levene’s test was used to test the equality of variances. Multivariate ANOVA was used to statistically test the differences between the three groups. In order to prevent data from incorrectly appearing to be statistically significant, the Bonferroni correction test was used. Moreover, we used the post hoc test in data analyses. 

To compare the means of the same variable between two groups, we used an independent group *t*-test. The *p*-value was set at 0.05.

## 3. Results

### 3.1. Pain Assessment Using VAS

**Figure 3:**
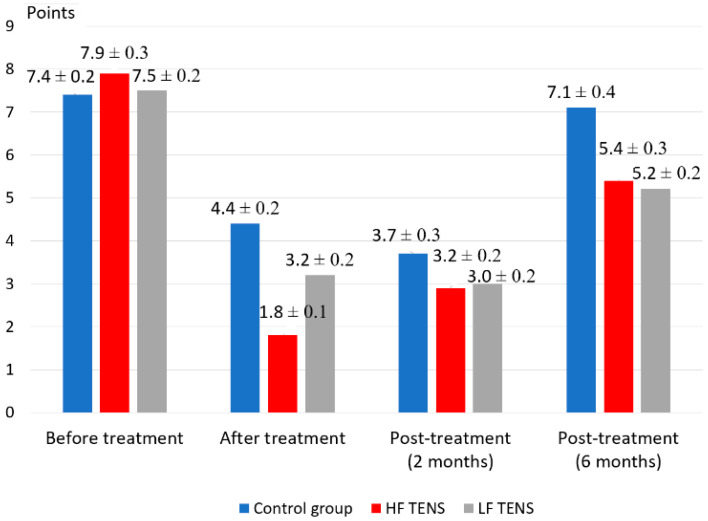
Dynamics of VAS pain assessment in diabetic patients with painful distal axonal neuropathy (mean ± S.E.M.).

Before treatment, mean pain intensity in TENS and control groups was 7.68 ± 0.14 points (Figure 3). After treatment, there was a significant decrease in pain intensity from baseline pre-treatment scores in both the control (40.5%, *p* < 0.05) and TENS groups (HF TENS group—77.2%, *p* < 0.05; LF TENS group—57.3%, *p* < 0.05). The intensity of pain 2 months after treatment did not significantly differ from the scores obtained immediately after treatment in the control and LF TENS groups (*p* > 0.05). The HF TENS group recorded a significant increase in pain intensity by 61.1%, *p* < 0.05.

After six months of the follow-up period, pain intensity did not significantly differ in the control group compared to baseline values before treatment. At the same time, pain intensity decreased in comparison to pre-treatment values with the use of HF TENS by 31.6%, *p* < 0.05 and LF TENS by 30.7%, *p* < 0.05. No significant difference in pain values between HF TENS and LF TENS groups was detected (*p* > 0.05). 

### 3.2. Pain Assessment Using MPQ

As is shown in Table 1, the mean total of PRI and mean PRI of sensory, affective and evaluative dimensions before treatment in the control and TENS groups were similar (*p* > 0.05). 

In the immediate post-treatment period, a significantly lower PRI was noted after TENS compared with the control group: in sensory dimensions by 32.0% (*p* < 0.05); in affective dimensions by 35.3% (*p* < 0.05); in evaluative dimensions by 52.9% (*p* < 0.05); in total PRI by 36.4% (*p* < 0.05).

Sensory dimension PRI immediately after treatment was lower, by 35.2% after HF TENS compared to LF TENS (*p* < 0.05), but did not differ after 2 and 6 months of follow-up periods (*p* > 0.05). Affective dimension PRI with the use of LF TENS was lower than HF TENS by 34.4% immediately after treatment (*p* < 0.05), by 47.3% after 2 months (*p* < 0.05) and by 34.8% after 6 months of follow-up period (*p* < 0.05). PRI differences in the evaluative dimension immediately after treatment were not statistically significant between HF TENS and LF TENS groups and did not differ between two TENS groups after 6 months of the follow-up period (*p* > 0.05). After 2 months of follow-up period, evaluative dimensions of PRI were higher in the HF TENS group than in the LF TENS group by 53.3% (*p* < 0.05).

Mean total PRI was significantly lower in TENS groups compared with control group immediately after treatment by 36.4% (*p* < 0.05), after 2 months of follow-up period by 35.6% (*p* < 0.05) and after 6 months of follow-up period by 33.5% (*p* < 0.05). Comparing total means of two groups, HF TENS and LF TENS, shows that pain value in the HF TENS group was lower than LF TENS by 12.4% immediately after treatment (*p* < 0.05) and was lower in LF TENS than in the HF TENS group by 20.7% after 2 months of the follow-up period (*p* < 0.05) and did not differ between two TENS groups after 6 months of the follow-up period (*p* > 0.05). 

### 3.3. Pain Assessment Using DN4

**Figure 4:**
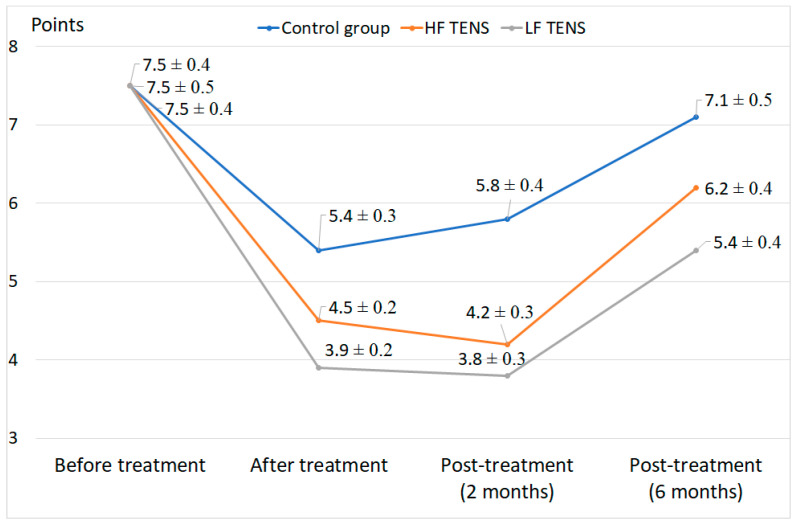
DN4 pain assessment during treatment of diabetic patients with painful distal axonal neuropathy at different periods (mean ± S.E.M.).

The sum of DN4 exceeded 4 and averaged 7.52 ± 0.07 points in all enrolled patients with diabetic painful distal axonal neuropathy. Complete resolution of neuropathic pain was observed in 21.5% of patients who underwent HF TENS and LF TENS, *p* < 0.05. In patients who received only standard drug therapy, complete resolution was not observed at all (Figure 4). 

The severity of neuropathic pain significantly decreased after HF TENS and LF TENS compared to drug therapy by 56.3%, *p* < 0.05. Comparing the dynamics of neuropathic pain between HF TENS and LF TENS, it can be noted that DN4 scores in the former decreased by more than 21% in comparison to the latter immediately after treatment, *p* < 0.05. The trend was maintained at 6 months of follow-up period with mean pain scores 14.8% higher in the LF TENS group, *p* < 0.05.

### 3.4. Pain Assessment Using Pain Drawing

**Figure 5:**
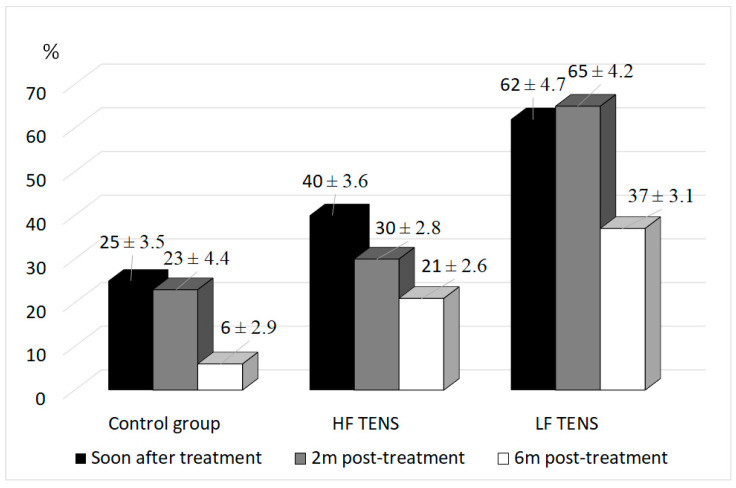
Pain projection area regression during treatment of diabetic patients with painful distal axonal neuropathy at different periods (mean ± S.E.M.).

In the control group, a decrease in PPA was registered by 25% immediately after treatment (*p* < 0.05) and 23% after 2 months of follow-up period (*p* < 0.05). At the 6-month mark PPA returned to pre-treatment baseline levels (*p* > 0.05). Figure 5 demonstrates that application of TENS decreased PPA by 51% immediately after treatment (*p* < 0.05), 47.5% after 2 months (*p* < 0.05) and 29% after 6 months of follow-up period (*p* < 0.05).

In comparison to the control group, immediate post-treatment PPA in the TENS group was two times less, *p* < 0.05. It is important to underline that decrease in PPA in the LF TENS group compared to HF TENS was 22% more immediately after treatment (*p* < 0.05), 35% more after 2 months of the follow-up period (*p* < 0.05) and 16% more after 6 months of the follow-up period (*p* < 0.05). 

Of note is the procedure’s safety, with no serious adverse events occurring during or after treatment [14].

## 4. Discussion

Neuropathic pain develops in 10–50% of patients with distal lower limb polyneuropathy in type 2 diabetes mellitus [15,16,17]. Almost all patients with diabetic neuropathic pain report quality of life impairment [18], with 80% experiencing poor sleep [19,20,21]. Unfortunately, drug therapy alone has not been effective enough in the treatment of many patients with severe neuropathic pain [18,22]. This has led to a necessity of increasing neuropathic pain pharmacotherapy efficacy through non-drug interventions [23]. The Toronto Expert Panel on Diabetic Neuropathy consensus recommends TENS as an effective non-pharmacological treatment for neuropathic pain [24].

Our results demonstrate that the use of TENS significantly enhances the analgesic effect of drug therapy as confirmed by multiple validated pain scales: VAS, MPQ, DN4 and Pain Drawing. The same results were obtained by other authors when studying the regression of pain syndrome using VAS [25] and MPQ [26]. In the literature, we did not find an application for DN4 and Pain Drawing to assess the dynamics of neuropathic pain using TENS. The findings suggest that HF TENS has a more pronounced analgesic effect than LF TENS in the immediate post-treatment period by 25.8% in VAS scores, 35.5% and 58.1% in MPQ evaluative and sensory dimensions, respectively, and by 21% in DN4. On the other hand, LF TENS had more analgesic effect than HF TENS in affective dimensions of MPQ by 51.3% and in Pain Drawing by 22%.

There are significant differences between HF TENS and LF TENS when post-treatment pain intensity is measured by multiple pain assessment tools. This reflects the distinctive effects of different TENS modalities on different aspects of neuropathic pain. The analgesic effect of HF TENS primarily addresses the sensory aspect of pain as a result of segmental gelatinous substance cell excitation, which has an inhibitory effect on nociceptive afferentation [27,28]. At the same time, long-term inhibition of nociceptive afferentation further enhances the segmental analgesic effect by reducing dorsal horn neuron sensitization [29,30,31]. Simultaneously there is a moderate secondary effect on the affective aspect of neuropathic pain as a consequence of decreased afferentation along nociceptive fibers. 

The therapeutic effect of LF TENS is probably achieved through two mechanisms:

Parasegmental mechanism: Parasegmental stimulation of the midbrain periaqueductal gray and rostral ventromedial medulla activates descending pain inhibitory pathways and inhibits descending facilitatory pain pathways [32,33,34]. Moreover, electrical stimulation of the central nervous system by LF TENS promotes the release of endorphins, which enhance the analgesic effect [32,33,34].

Local mechanisms: In many clinical and experimental studies, LF TENS was found to be highly effective in the treatment of distal polyneuropathy due to improving microcirculation and activating reparative processes in affected nerves [2,6,7,8,9]. 

**Figure 6:**
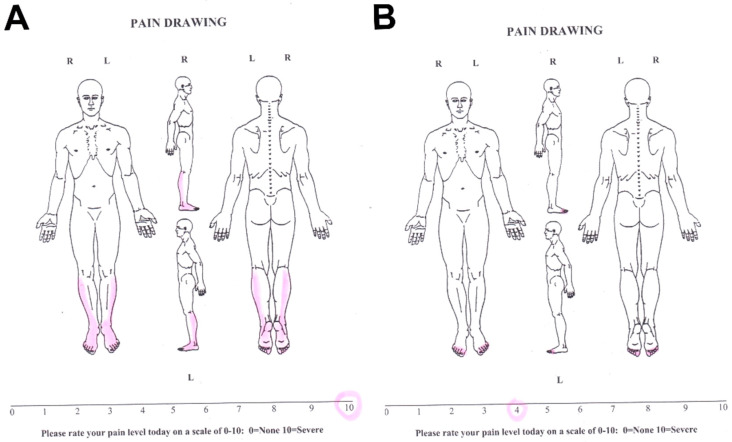
The severity of pain syndrome determined using Pain Drawing and VAS before (**A**) and after treatment (**B**) of a patient with severe neuropathic pain syndrome. Pain projection area before treatment was 1550 mm2 and regressed after treatment to 125 mm2. Thus, the regression of pain syndrome according to Pain Drawing was 91.9%, but according to VAS, it was only 60%.

It is important to note a stronger correlation between pain projection and affective rather than sensory pain components. The drawing pain projection reflects not just sensory disorders and pain locations. However, patients’ emotional reaction to pain is also very important; patients can depict pain subjectively more than in the affected area in nature. Some authors found a weak correlation between Pain Drawing pain syndrome severity and the patient’s depressive state (Figure 6) [35]. 

In our study analgesic effect is prolonged and maintained 6 months after completion of TENS treatment. The maximum analgesic effect in the post-treatment period was recorded 2 months after TENS, with gradual regression over the next 4 months. Warke et al. demonstrated that the long-term analgesic effect of low-frequency TENS could last for about 7 months [36], while Nabi and Jin reported a 3 months effect following the application of high-frequency TENS [31,37].

Our findings, supported by the existing literature, suggest that this prolonged analgesic effect is due to two factors. Firstly, regression of central sensitization, with a delay in its formation while maintaining nociceptive afferentation [38,39,40,41]. Secondly, improvements in damaged peripheral nerve condition with decreased nociceptive afferentation and increased sensory afferentation. In the best-case scenario, this mechanism prevents the development of central sensitization or, in the least, slows down the process of its formation [10,11,13,37]. 

As a result of this study, we recommend:

- Using direct TENS of peroneal and tibial nerves in treatment of neuropathic pain;
- A combination of HF TENS and LF TENS in treatment of patients with neuropathic pain;
- Complex application of VAS, MPQ, DN4 and Pain Drawing in neuropathic pain diagnosis in patients with diabetic neuropathies.

However, these guidelines can be changed in other countries and other racial groups of patients, depending on the genetic and nongenetic (cultural, religious, ethnicity, etc.) factors that influence pain perception.

## 5. Limitations

- It was not possible to study the effectiveness of the combined use of HF TENS and LF TENS in the treatment of diabetic neuropathic pain and to compare these results with the use of separate methods of TENS;
- There was no comparative analysis of the effectiveness of HF TENS and LF TENS in the treatment of diabetic neuropathic pain between patients with type 1 diabetes mellitus and type 2 diabetes mellitus;
- We could not investigate the effectiveness of TENS methods in the treatment of patients with myelinopathy type of peripheral nerve disorders, and we could not compare these results with the results of treatment of patients with an axonal disorder;
- We could not study the effectiveness of TENS compared with the use of sham stimulation in the control group.

## 6. Conclusions

There are significant differences between HF and LF TENS based on pain assessment using various pain scales, which reflects the distinctive effects of different TENS modalities on different aspects of pain. Sensory aspects of pain respond better to HF TENS therapy and affective aspects to LF TENS.

## Figures and Tables

**Table 1:** Dynamics of pain rating index of sensory and affective dimensions of MPQ during treatment of diabetic patients with painful distal axonal neuropathy at different periods.

Group	Before Treatment (Mean ± S.E.M.)	After Treatment (Mean ± S.E.M.)	2 Months Post-treatment (Mean ± S.E.M.)	6 Months Post-treatment (Mean ± S.E.M.)
Sensory Dimensions				
Control group	16.2 ± 0.41	11.4 ± 0.56 *	10.4 ± 0.40 *	14.7 ± 0.49
HF TENS	15.3 ± 0.42	6.10 ± 0.43 *#	7.22 ± 0.43 *#	10.3 ± 0.36 *#
LF TENS	15.2 ± 0.43	9.42 ± 0.42 *#	7.51 ± 0.42 *#	11.1 ± 0.45 *#
Affective Dimensions				
Control group	10.2 ± 0.39	6.4 ± 0.28 *	7.2 ± 0.27 *	9.82 ± 0.4
HF TENS	9.10 ± 0.35	5.0 ± 0.21 *#	5.5 ± 0.11 *#	6.9 ± 0.29 *#
LF TENS	10.3 ± 0.36	3.28 ± 0.27 *#	2.9 ± 0.17 *#	4.5 ± 0.34 *#
Evaluative Dimensions				
Control group	4.5 ± 0.11	3.4 ± 0.26 *	3.3 ± 0.20 *	4.3 ± 0.19
HF TENS	4.6 ± 0.12	1.5 ± 0.13 *#	2.3 ± 0.13 *#	2.8 ± 0.16 *#
LF TENS	4.6 ± 0.11	1.7 ± 0.12 *#	1.5 ± 0.12 *#	2.7 ± 0.15 *#
Total Pain Rating Index				
Control group	30.9 ± 0.37	21.2 ± 0.25 *	20.9 ± 0.23 *	28.8 ± 0.24
HF TENS	29.0 ± 0.39	12.6 ± 0.24 *#	15 ± 0.22 *#	20 ± 0.24 *#
LF TENS	30.1 ± 0.37	14.4 ± 0.24 *#	11.9 ± 0.17 *#	18.3 ± 0.23 *#

Note: * *p* < 0.05—reliability of differences in results compared to the initial values before treatment; # *p* < 0.05—compared with similar values of the control group.

## References

[1] Al Zamil M.K. (2019). Results of a comparative analysis between transcutaneous electroneurostimulation and acupuncture in the treatment of 548 patients with diabetic distal polyneuropathy of the lower extremities. Clin. Neurol..

[2] Gürgen S.G., Sayın O., Cetin F., Tuç Yücel A. (2014). Transcutaneous electrical nerve stimulation (TENS) accelerates cutaneous wound healing and inhibits proinflammatory cytokines. Inflammation.

[3] Vieira P.J., Ribeiro J.P., Cipriano G., Umpierre D., Cahalin L.P., Moraes R.S., Chiappa G.R. (2012). Effect of transcutaneous electrical nerve stimulation on muscle metaboreflex in healthy young and older subjects. Eur. J. Appl. Physiol..

[4] Haan N., Song B. (2014). Therapeutic application of electric fields in the injured nervous system. Adv. Wound Care.

[5] Kutlu A.K., Ceçen D., Gürgen S.G., Sayın O., Cetin F. (2013). A Comparison Study of Growth Factor Expression following Treatment with Transcutaneous Electrical Nerve Stimulation, Saline Solution, Povidone-Iodine, and Lavender Oil in Wounds Healing. Evid Based Complement. Alternat. Med..

[6] Luo B., Huang J., Lu L., Hu X., Luo Z., Li M. (2014). Electrically induced brain derived neurotrophic factor release from Schwann cells. J. Neurosci. Res..

[7] Forciniti L., Ybarra J., Zaman M., Schmidt C. (2014). Schwann cell response on polypyrrole substrates upon electrical stimulation. Acta Biomater..

[8] Al-Majed A.A., Neumann C.M., Brushart T.M., Gordon T. (2000). Brief electrical stimulation promotes the speed and accuracy of motor axonal regeneration. J. Neurosci..

[9] Asensio-Pinilla E., Udina E., Jaramillo J., Navarro X. (2009). Electrical stimulation combined with exercise increase axonal regeneration after peripheral nerve injury. Exp. Neurol..

[10] Al Zamil M., Kulikova N., Bezrukova O., Volkova I., Stahurlova V. (2019). Effectiveness of transcutaneous electrical neurostimulation for treatment of diabetic distal polyneuropathy. Eur. J. Neurol..

[11] Al Zamil M.K., Kulikova N.G., Minenko I.A., Vasil’va E.S. (2020). Direct transcutaneous electroneurostimulation in the treatment of pathologies of the peripheral nervous system. Physiotherapist.

[12] Eberstein A., Eberstein S. (1996). Electrical stimulation of denervated muscle: Is it worth, while?. Med. Sci. Sports Exerc..

[13] Gibson W., Benedict M., Wand B.M., Meads C., Catley M.J., O’Connell N.E. (2019). Transcutaneous electrical nerve stimulation (TENS) for chronic pain—An overview of Cochrane Reviews. Cochrane Database Syst. Rev..

[14] Nabi B.N., Saberi A., Eghbali B.B., Hosseininezhad M., Gelareh Biazar G. (2021). Efficacy and Safety of TENS and Duloxetine in Patients with Painful Diabetic Neuropathy: A Single Blind Randomized Clinical Trial. J. Adv. Med. Biomed. Res..

[15] Petrova M.M., Shnayder N.A., Kirichkova G.A. (2008). Diabetic neuropathy: Definition, epidemiology. Sib. Med. Rev..

[16] Didangelos T., Doupis J., Veves A. (2014). Painful diabetic neuropathy: Clinical aspects. Handb. Clin. Neurol..

[17] Tesfaye S., Boulton A.J., Dickenson A.H. (2013). Mechanisms and management of diabetic painful distal symmetrical polyneuropathy. Diabetes Care.

[18] Davoudi M., Rezaei P., Rajaeiramsheh F., Ahmadi S., Taheri A.A. (2021). Predicting the quality of life based on pain dimensions and psychiatric symptoms in patients with Painful diabetic neuropathy: A cross-sectional prevalence study in Iranian patients. Health Qual Life Outcomes.

[19] Melikoglu M.A., Celik A. (2017). Does Neuropathic Pain Affect the Quality of Sleep?. Eurasian J. Med..

[20] Minenko I.A., Al-Zamil M.K. (2017). Dynamics of the quality of life of patients with diabetic neuropathic pain syndrome against the background of the complex application of transcutaneous electrical nerve stimulation and acupuncture. Sib. Sci. Med. J..

[21] Singh-Franco D., Jacobs R.J. (2017). Patient perspectives on peripheral neuropathic pain experience within the community. Diabetes Metab. Syndr. Clin. Res. Rev..

[22] Teixeira M.J. (2009). Challenges in the treatment of neuropathic pain. Drugs Today.

[23] Moisset X., Bouhassira D., Couturier J.A., Alchaar H., Conradi S., Delmotte M.H., Lanteri-Minet M., Lefaucheur J.P., Mick G., Piano V. (2020). Pharmacological and non-pharmacological treatments for neuropathic pain: Systematic review and French recommendations. Rev. Neurol..

[24] Tesfaye S., Vileikyte L., Rayman G., Sindrup S.H., Perkins B.A., Baconja M., Vinik A.I., Boulton A.J.M. (2011). Painful diabetic peripheral neuropathy: Consensus recommendations on diagnosis, assessmentand management. Diabetes Metab Res. Rev..

[25] Shahanawaz S.D. (2014). Effect of high TENS on neuropathic pain in diabetic neuropathy patients. Int. J. Physiother. Res..

[26] Upton G.A., Tinley P., Al-Aubaidy H., Crawford R. (2017). The influence of transcutaneous electrical nerve stimulation parameters on the level of pain perceived by participants with painful diabetic neuropathy: A crossover study. Diabetes Metab Syndr..

[27] Melzack R., Wall P.D. (1984). Acupuncture and transcutaneous electrical nerve stimulation. Postgrad Med. J..

[28] Melzack R., Wall P.D. (1965). Pain mechanisms: A new theory. Science.

[29] Sabino G.S., Santos C.M., Francischi J.N., Resende M.A. (2008). Release of endogenous opioids following transcutaneous electric nerve stimulation in an experimental model of acute inflammatory pain. J. Pain.

[30] Gozani S.N. (2019). Remote Analgesic Effects of Conventional Transcutaneous Electrical Nerve Stimulation: A Scientific and Clinical Review With A Focus On Chronic Pain. Pain Res..

[31] Nabi B.N., Sedighinejad A., Haghighi M., Biazar G., Hashemi M., Haddadi S., Fathi A. (2015). Comparison of transcutaneous electrical nerve stimulation and pulsed radiofrequency sympathectomy for treating painful diabetic neuropathy. Anesthesiol. Pain Med..

[32] Johnson M. (2007). Transcutaneous Electrical Nerve Stimulation: Mechanisms, Clinical Application and Evidence. Rev. Pain.

[33] Kocyigit F., Akalin E., Gezer N.S., Orbay O., Kocyigit A., Ada E. (2012). Functional magnetic resonance imaging of the effects of low-frequency transcutaneous electrical nerve stimulation on central pain modulation: A double-blind, placebo-controlled trial. Clin. J. Pain.

[34] Mokhtari T., Ren Q., Li N., Wang F., Bi Y., Hu L. (2020). Transcutaneous Electrical Nerve Stimulation in Relieving Neuropathic Pain: Basic Mechanisms and Clinical applications. Curr. Pain Headache Rep..

[35] Reis F., Guimarães F., Nogueira L.C., Meziat-Filho N., Sanchez T.A., Wideman T. (2019). Association between pain drawing and psychological factors in musculoskeletal chronic pain: A systematic review. Physiother. Theory Pract..

[36] Warke K., Al-Smadi J., Baxter D. (2006). Walsh, D.M. Lowe-Strong AS. Efficacy of transcutaneous electrical nerve stimulation (TENS) for chronic low-back pain in a multiple sclerosis population: A randomized, placebo-controlled clinical trial. Clin. J. Pain.

[37] Jin D., Xu Y., Geng D., Yan T. (2010). Effect of transcutaneous electrical nerve stimulation on symptomatic diabetic peripheral neuropathy: A meta-analysis of randomized controlled trials. Diabetes Res. Clin. Pract..

[38] Sluka K.A., Bjordal J.M., Marchand S., Rakel B.A. (2013). What makes transcutaneous electrical nerve stimulation work? Making sense of the mixed results in the clinical literature. Phys. Ther..

[39] Sluka K.A., Deacon M., Stibal A., Strissel S., Terpstra A. (1999). Spinal blockade of opioid receptors prevents the analgesia produced by TENS in arthritic rats. J. Pharmacol. Exp. Ther..

[40] Somers D., Clemente F.R. (2006). Transcutaneous electrical nerve stimulation for the management of neuropathic pain: The effects of frequency and electrode position on prevention of allodynia in a rat model of complex regional pain syndrome type II. Phys. Ther..

[41] Vance C.G., Dailey D.L., Rakel B.A., Sluka K.A. (2014). Using TENS for pain control: The state of the evidence. Pain Manag..

